# Increased TIGIT expression correlates with impaired NK cell function in diffuse large B-cell lymphoma

**DOI:** 10.3389/fonc.2025.1551061

**Published:** 2025-03-31

**Authors:** Hui Lu, Xiaoyan Zhao, Liqiong Liu, Lu Zhang, Huafang Wang

**Affiliations:** ^1^ Department of Hematology, Union Hospital, Tongji Medical College, Huazhong University of Science and Technology, Wuhan, China; ^2^ Department of Pediatrics, Tongji Hospital, Tongji Medical College, Huazhong University of Science and Technology, Wuhan, China; ^3^ Department of Hematology, Shenzhen Nanshan People’s Hospital and the 6th Affiliated Hospital of Shenzhen University Health Science Center, Affiliated Shenzhen Sixth Hospital of Guangdong Medical University, Shenzhen, China

**Keywords:** DLBCL, NK cells, CD56 dim NK cells, TIGIT, CD226

## Abstract

**Purpose:**

This study aims to investigate the status of natural killer (NK) cells and the role of T-cell immunoreceptor with Ig and ITIM domains (TIGIT)-mediated regulation in diffuse large B-cell lymphoma (DLBCL).

**Methods:**

Peripheral blood samples from 30 newly diagnosed DLBCL patients and 25 healthy controls were collected. Multiparametric flow cytometry was used to analyze the expression levels of TIGIT and its family molecules (CD226 and CD96) on NK cells, as well as to assess NK cell phenotype and function. The restorative effects of TIGIT blockade on NK cell cytotoxicity were evaluated through *in vitro* functional assays and *in vivo* animal models.

**Results:**

Compared to healthy controls, DLBCL patients exhibited significantly reduced percentages and absolute numbers of NK cells. TIGIT expression was markedly upregulated on NK cells in DLBCL patients, while CD226 expression was downregulated; however, no significant difference in CD96 expression was observed. These alterations were associated with impaired NK cell function in DLBCL patients, including reduced secretion of activation factors such as granzyme B, perforin, and CD107a. Importantly, TIGIT blockade significantly enhanced the cytotoxic activity of NK cells against DLBCL cells in both *in vitro* and *in vivo* settings.

**Conclusion:**

Dysregulated expression of TIGIT and its family molecules on NK cells contributes to NK cell dysfunction and promotes tumor immune escape in DLBCL. These findings highlight TIGIT as a promising therapeutic target for restoring NK cell-mediated antitumor immunity in DLBCL.

## 1 Introduction

As a highly aggressive subtype of Non-Hodgkin’s lymphoma (NHL), diffuse large B-cell lymphoma (DLBCL) exhibits the highest incidence rate within NHL, accounting for 30 - 40% of cases (1). Notably, in Asian countries, the incidence rate reaches as high as 50% (2). DLBCL exhibits significant heterogeneity in clinical manifestations and prognostic outcomes among patients (3). Currently, chemotherapy remains the cornerstone of DLBCL treatment, with the R-CHOP regimen (rituximab combined with cyclophosphamide, doxorubicin, vincristine and prednisolone) serving as the standard first-line therapy. While approximately 60% of patients achieve complete remission, the remaining patients suffer from refractory or relapsed disease due to R-CHOP resistance, resulting fare poorly (4, 5). According to the international SCHOLAR-1 study, the median overall survival for patients refractory to first-line treatment is only 6.3 months (6).

Studies have identified distinct differences in gene and protein expression within the tumor microenvironment between R-CHOP-sensitive and R-CHOP-resistant DLBCL patients (7). Analysis of 405 DLBCL cases revealed that a lack of T cells and/or NK cells, along with increased programmed cell-death 1 (PD-1) expression on immune cells, is associated with poor prognosis and correlates with the resistance to standard immunochemotherapy (8, 9). The poor prognosis associated with R-CHOP resistance has driven the exploration of novel therapeutic strategies for DLBCL. Emerging immunotherapies, such as chimeric antigen receptor-T cell (CAR-T) therapy, adoptive natural killer (NK) cell therapy, and immune checkpoint inhibitors, offer new avenues for immunotherapy and the discovery of novel therapeutic targets in DLBCL treatment (10, 11).

Exploring the immune status of DLBCL patients thus holds significant potential for identifying novel immunotherapeutic targets. As key components of the innate immune system, NK cells play a critical role in maintaining immune homeostasis (12). Human NK cells, characterized by a CD3^-^CD56^+^ immunophenotype, can be further classified into two subtypes based on CD56 expression: CD56^dim^CD16^bright^NK cells, which represent a mature and highly cytotoxic population, and CD56^bright^CD16^low/-^NK cells, a less mature cell subset with immunoregulatory functions (13). CD56^dim^CD16^bright^NK cells account for approximately 90% of peripheral NK cells and mediate antibody-dependent cell-mediated cytotoxicity (14). The precise regulation of NK cell activity relies on a dynamic balance between activating and inhibitory receptors on their surface. Under normal physiological conditions, NK cells interact with MHC-expressing cells through inhibitory receptors, suppressing the downstream effects of activating receptors and protecting normal cells from NK-mediated cytotoxicity (15). However, NK cells uniquely recognize and eliminate target cells in an MHC-unrestricted manner, enabling them to effectively target tumor cells that often downregulate MHC expression as an immune evasion strategy. Upon recognition abnormal cells, NK cells activate their effector functions through activating receptors, releasing cytokines to modulate immune responses and directly mediating cytotoxicity (16). In DLBCL, NK cell dysfunction contributes to tumor immune evasion, underscoring the importance of elucidating the mechanisms regulating NK cell activity in this context.

Immune evasion is a hallmark of DLBCL, driven in part by the upregulation of immune checkpoint molecules. Currently, PD-1 and cytotoxic T-lymphocyte-associated antigen 4 (CTLA-4) are two well-characterized and extensively studied in the DLBCL tumor immune microenvironment (8). Notably, T-cell immunoglobulin mucin-3 (Tim-3) expression levels correlate closely with the Ann-Arbor staging of DLBCL, whereas lymphocyte activation gene 3 (LAG-3) overexpression is strongly associated with poor clinical outcomes (17). Recent studies have reported that blocking inhibitory immune checkpoints, such as PD-1 and CTLA-4, can enhance antitumor immunity and restore immune responses in various malignancies (18). However, the therapeutic efficacy of PD-1 inhibitors remains limited in unselected DLBCL patient populations, highlighting the need for novel combination therapies (19). Therefore, investigating the expression profiles and functional roles of novel immune checkpoint molecules in DLBCL patients is critical for advancing diagnostic approaches and optimizing combination treatments.

T-cell immunoreceptor with Ig and ITIM domains (TIGIT) is a novel inhibitory immune checkpoint molecule composed of three structural domains: an extracellular region, a transmembrane region, and a cytoplasmic region. TIGIT is primarily expressed on NK cells, CD8^+^ T cells, CD4^+^ T cells, and regulatory T cells (Tregs) (20–24). By binding to its ligands, CD155 and/or CD122, TIGIT suppresses immune cell function, promoting tumor immune evasion (25). TIGIT, along with its family members CD226 (DNAM-1) and CD96 (TACTILE), competes for shared ligands, with TIGIT transmitting co-inhibitory signals and CD226 delivering co-stimulatory signals. CD96 contains both an ITIM motif, which transmits inhibitory signals, and a YXXM motif, which facilitates receptor activation (26, 27). Elevated TIGIT expression has been observed on lymphoma-infiltrating T cells in HL and certain subtypes of NHL, including DLBCL (28). Previous studies have also characterized the expression profiles of TIGIT and its family molecules on NK cells in other hematologic malignancies, such as myelodysplastic syndromes (MDS) and acute myeloid leukemia (AML) (29, 30). However, the expression patterns of TIGIT and its family members on NK cells, as well as their functional impact on NK cell activity in DLBCL, remains poorly understood. Furthermore, the therapeutic potential of TIGIT blockade in restoring NK cell function in DLBCL has not been systematically explored, highlighting the need for further investigation in this context.

To address these gaps, our study aims to comprehensively evaluate the changes in expression levels of TIGIT and its family molecules on NK cells in DLBCL patients and to investigate their functional impact on NK cell-mediated immunity. This findings will advance the understanding of immune checkpoint regulation in DLBCL and provide critical insights into the role of TIGIT in modulating NK cell biology. By elucidating the mechanisms underlying NK cell dysfunction in DLBCL, our findings will not only deepen the understanding of the immune landscape in these patients, but also lay the groundwork for the development of novel immunotherapies targeting TIGIT and its associated pathways.

## 2 Materials and methods

### 2.1 Patients

The peripheral blood from 30 patients (18 males and 12 females) with newly diagnosed DLBCL and 25 age-matched healthy controls (HCs) (13 males and 12 females) were collected from the Hematology Department of Union Hospital affiliated to Tongji Medical College, Huazhong University of Science and Technology. Peripheral blood samples were collected prior to the initiation of standard treatment. Basic characteristics of all patients with DLBCL included in our study are summarized in **Table 1**. HCs were critically selected on the basis of clinical records and laboratory examinations. Participants were excluded from the study if they had severe dysfunction of the heart, liver, or kidneys, primary malignant tumors of other systems, acute infectious diseases, or autoimmune diseases. All participants in this study were consecutively recruited to minimize the potential for selection bias. The study was conducted in accordance with the Declaration of Helsinki and was approved by the Ethics Committee of Union Hospital, Tongji Medical College, Huazhong University of Science, and Technology.

**Table 1:** DLBCL patient characteristics.

Variable	Number	Percentage(%)
**Age**	45(19-74)	
Gender		
Male	18	60
Female	12	40
Site of Involvement		
Within Lymph Nodes	23	76.7
Outside Lymph Nodes	7	23.3
Ann Arber Stage^*^		
I-II	14	46.7
III-IV	16	53.3
Cellular Origin Type^**^		
GCB type	10	33.3
non-GCB type	20	66.7
LDH		
≤245U/L	17	56.7
>245U/L	13	43.3
IPI score^***^		
0-2	20	66.7
3-5	10	33.3

^*^ The Ann Arbor staging system was used. Stage I indicates disease confined to a single lymph node region or one extranodal site. Stage II involves two or more lymph node regions on the same side of the diaphragm, possibly including a localized extranodal site. Stage III signifies disease on both sides of the diaphragm, potentially involving spleen or other extranodal sites. Stage IV represents widespread or disseminated disease affecting multiple extranodal organs or tissues. ^**^ The classification was determined using the Hans algorithm, which is based on immunohistochemical expression of CD10, BCL6, and MUM1. Cases expressing CD10 or showing BCL6 positivity without MUM1 expression were classified as GCB type, while those that were CD10-negative and either MUM1-positive or BCL6-negative were classified as non-GCB type. ^***^ The IPI scoring system assesses five factors: age over 60, elevated serum LDH, ECOG performance status of 2-4, Ann Arbor stage III or IV, and involvement of more than one extranodal site. Each factor scores 1 point if present, with total scores ranging from 0 to 5.

Sample size calculation: The sample size estimation was performed using PASS 15 software, based on previous studies reporting mean TIGIT expression levels on NK cells in MDS patients and HCs (29). With a confidence level of 95% (1-α = 0.95) and a statistical power of 90% (1-β = 0.90), the calculated sample size was 13 per group. To enhance the reliability of our results, we increased the sample size to 30 DLBCL patients and 25 healthy controls in this study.

### 2.2 Cell culture

SU-DHL-4 cells were obtained from the China Center for Type Culture Collection (Wuhan, China). A20 cells were obtained from American Type Culture Collection (Manassas, USA). Cells were grown in RPMI 1640 Medium (Gibco, USA) supplemented with 10% fetal bovine serum (FBS; Gibco, USA), 100 U/ml of penicillin, and 100 μg/ml of streptomycin in a 5% CO_2_ humidified atmosphere at 37°C.

### 2.3 Peripheral blood NK cell counting

All peripheral blood samples were stored at 4°C immediately after collection and processing began within two hours of collection. To determine the absolute NK cells in peripheral blood, a flow cytometry-based method was employed. After adding 5 µL each of anti-human CD3-PE-Cy7 and anti-human CD16-APC-Cy7 antibodies, along with 20 µL of anti-human CD56-APC antibody, 100 µL of freshly collected peripheral blood was added to these tubes, followed by gentle vertexing to ensure thorough mixing. The samples were incubated at 4°C in the dark for 30 minutes to allow for antibody binding. For absolute counting, 500 µL of PBS was added to resuspend the cells after lysing the red blood cells. Then, 100 µL of pre-mixed 123 COUNT™ beads (eBioscience, USA) (vortexed before use to ensure even distribution) was added. Prior to analysis, each sample tube was vortexed again to ensure homogeneity.


$$\begin{array}{c} \text{Absolute Count}\left(\text{cells}/\text{µL}\right)\;=\;\left(\text{Cell Count}\right)/\left(\text{eBead Count}\right)\;\\ \times \text{eBead Concentration} \end{array}$$

(eBead Concentration in this study: 1010000 ebeads/mL)

### 2.4 Isolation of peripheral blood mononuclear cells

All peripheral blood samples were stored at 4°C immediately after collection and processing began within two hours of collection. The isolation of PBMCs begins with diluting the whole blood in an equal volume of PBS, followed by carefully layering this diluted blood over Ficoll-Paque solution (Cytiva, USA) in a centrifuge tube. After centrifugation at 400 g for 30 - 40 minutes without the brake to allow separation, the PBMC layer is collected from the interface between the plasma and the Ficoll-Paque. These cells are then washed by resuspending them in PBS. Then, the cell viability was evaluated using trypan blue staining (Beyotime, China), and ensured that the cell viability exceeded 95%.

### 2.5 Multiparametric flow cytometric analysis

For each specimen, three flow cytometry tubes were required. Tube 1: anti-human CD3-PE-Cy7, anti-human CD56-APC, anti-human CD16-APC-Cy-7, anti-human TIGIT-PerCP, anti-human PD-1-FITC, and anti-human CD226-PE. Tube 2: anti-human CD3-PE-Cy7, anti-human CD56-APC, anti-human CD16-APC-Cy-7, anti-human CD96-PE, anti-human granzyme B-FITC, and anti-human perforin-PerCP. Tube 3: anti-human CD3-PE-Cy7, anti-human CD56-APC, anti-human CD16-APC-Cy-7, anti-human CD107a-PE, and anti-human IFN-γ-PerCP. Single-stained controls were used to adjust compensation settings and to minimize spectral overlap between fluorochromes. Fluorescence-minus-one (FMO) controls and isotype controls were used to establishing accurate gating strategies, ensuring precise discrimination between positive and negative populations.

For surface staining, PBMCs were stained with fluorochrome-conjugated mAbs. The samples were incubated with antibodies at 4°C for 30 minutes. After that, they were washed with the staining buffer and stored at 4°C until the time of analysis.

For intracellular staining of granzyme B and perforin, after being fixed and permeabilized with Fixation/Permeabilization solution (BD Pharmingen, USA) for 20 min at 4°C, cells were washed in 1 × Perm/Wash buffer (BD Pharmingen, USA). Then, intracellular proteins were labeled with the corresponding fluorochrome-conjugated mAbs following the manufacturer’s instructions in 50 μL 1 × Perm/Wash buffer (BD Pharmingen, USA).

For intracellular staining of CD107a and IFN-γ, the cells were cultured in 48-well plates at a density of 2 × 10^6^/mL and were stimulated with Leukocyte Activation Cocktail (2 μL/mL) (BD Pharmingen, USA) for 6 h at 37°C with 5% CO_2_ for NK cell stimulation and protein transport inhibition. Then, samples were undergone surface staining and intracellular staining as described above. The list of all mAbs was presented in **Supplementary Table S1**. Flow cytometry was conducted on a BD LSRFortessa X-20, and the data were analyzed with FlowJo V10 software. Daily quality control checks were performed to verify the stability and sensitivity of the instrument.

### 2.6 Co-culture of purified NK cells and SU-DHL-4 cells

Peripheral NK cells were isolated and purified using Direct Human NK Cell Isolation Kit (Stemcell Technologies, Canada). The purity of the isolated NK cells was assessed using flow cytometry with anti-human CD3-PE-Cy7 and anti-human CD56-APC, with a purity exceeding 90%. Cell viability was evaluated using trypan blue staining (Beyotime, China), and the viability of the isolated NK cells was consistently greater than 95%. TIGIT^+^ NK cells were further purified by fluorescence-activated cell sorting (FACS). The isolated NK cells were then stimulated with IL-2 (100 U/mL, Peprotech), IL-12 (100 U/mL, Peprotech) for 72 h in IMDM complete medium. These cytokine concentrations were selected based on extensive optimization experiments and well-established protocols from the previous study (22). IL-12 is a key NK cell-stimulating factor that promotes proliferation, cytotoxicity, and cytokine production, while IL-2 amplifies the response of NK cells to IL-12 by upregulating the expression of the IL-12 receptor (31, 32).

SU-DHL-4 cells labeled with CFSE (BD Horizon, USA) were collected as target cells. SU-DHL-4 cells were co-cultured with purified NK cells at the effector cell: target cell ratios (E:T ratio) of 0:1, 5:1, 10:1 and 20:1 for 6 hours. These E:T ratios were chosen to cover a dynamic range of cytotoxic activity, ensuring physiological relevance and optimal experimental efficiency. After co-cultivation, 5ul DAPI solution (BD Pharmingen, USA) was used to detect the killing ability of NK cells. Following a 5-minute incubation, flow cytometry was performed to analyze the results. In some experiments, varying concentrations of anti-TIGIT mAb (5 μg/mL, 10 μg/mL, and 20 μg/mL; eBioscience, USA) or an IgG isotype control (10 μg/mL; R&D Systems, USA) were added to block TIGIT pathway.


$$\text{Killing effect }=\;\left(\text{Percentage of target cell death in the co-culture group }-\text{ Percentage of target cell spontaneous death}/\left(1\;-\text{ Percentage of target cell spontaneous death}\right)\right)\;\times \;100\%.$$

### 2.7 Mice and *in vivo* experiments

Rag2-/- mice were purchased from Cyagen Biosciences. The mice were housed in a pathogen-free facility at the Animal Laboratory Center of Huazhong University of Science and Technology. All animal experimental procedures and protocols involving animals were approved by the Animal Care Committee of Tongji Medical College, Huazhong University of Science and Technology, and in accordance with the Guide for the Care and Use of Laboratory Animals promulgated by the National Institutes of Health.

A20 cells (1 × 10^6^ cells per mouse) were subcutaneously implanted into the flanks of the mice. For anti-TIGIT blockade therapy, 200μg of anti-TIGIT mAb (clone IG9; BioX Cell, USA) was injected intraperitoneally into the mice on days 3, 6, 9, 12. Primary tumor volume was measured once on days 4, 7, 10, 13, and 16 using calipers and calculated according to the following formula: tumor volume (mm^3^) = (longest diameter × shortest diameter^2^)/2.

### 2.8 Statistical analysis

FlowJo V10 software was used to analyze all flow data. All statistical analyses were performed using GraphPad Prism software (version 8.0.2, USA). Student’s t-test or two-way analysis of variance was used to evaluate statistical differences, as appropriate. Pearson test was used for correlation analysis. All data are presented as mean  ±  standard deviation (SD). A P value of < 0.05 was considered statistically significant.

## 3 Results

### 3.1 Abnormal TIGIT and its family molecules expression on NK cells from patients with DLBCL

**Figure 1:**
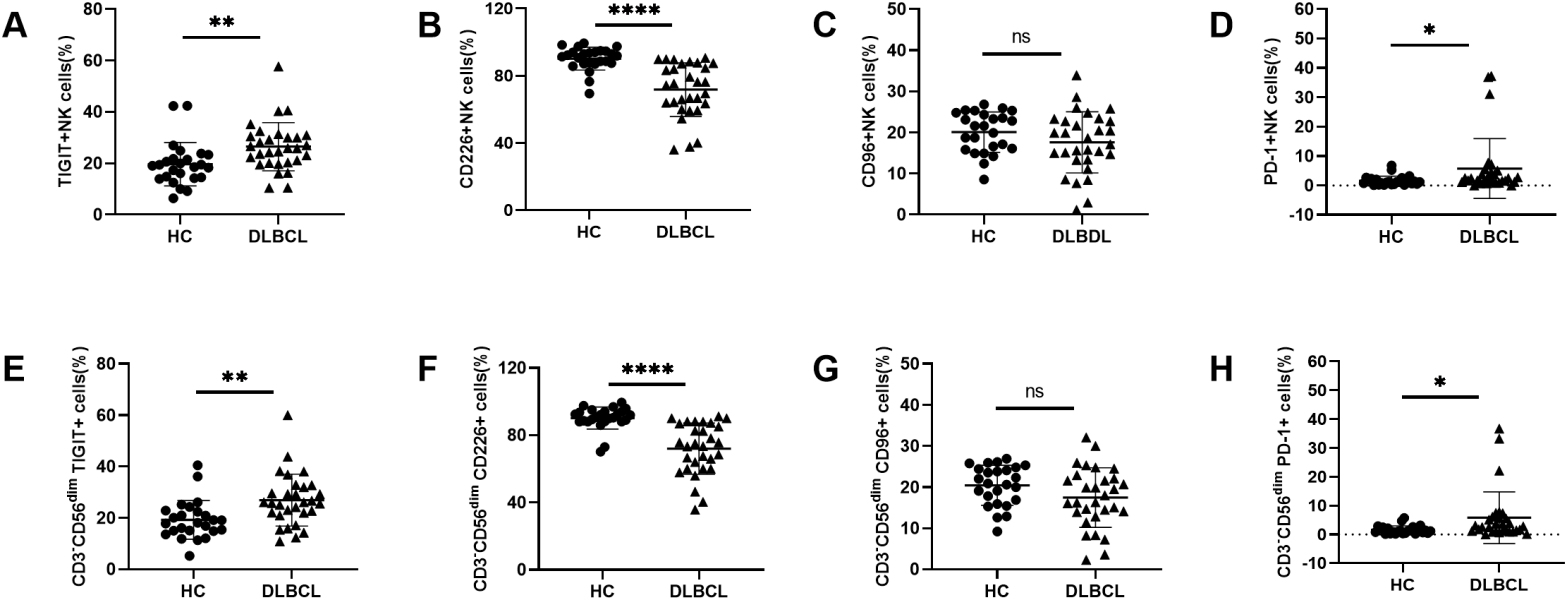
Abnormal TIGIT and its family molecules expression on NK cells from DLBCL patients **(A–D)** Comparisons of expression levels of TIGIT, CD226, CD96, and PD-1 on NK cells from DLBCL patients and HCs. **(E–H)** Comparisons of expression levels of TIGIT, CD226, CD96, and PD-1 on CD3^-^CD56^dim^NK cells from DLBCL patients and HCs. Each symbol represents the mean value from three technical replicates of one subject, and the data are shown as mean ± SD. HC, healthy control, DLBCL, diffuse B-cell lymphoma. DLBCL (n=30), HC (n=25). * indicates P<0.05, ** indicates P<0.01, **** indicates P<0.0001, ns indicates no significance. Unpaired Student’s t-test was used.

We firstly evaluated the frequency and subsets of NK cells in peripheral blood of HCs and DLBCL patients (**Supplementary Figure S1A**). The frequency and subsets of NK cells in peripheral blood were evaluated using flow cytometry. Compared to HCs, DLBCL patients exhibited significantly lower percentages and absolute numbers of NK cells, particularly CD56^dim^ NK cells. (**Supplementary Figure S2**). We further analyzed the expression of TIGIT and its family molecules, including TIGIT, CD226, CD96 and PD-1, on NK cells from DLBCL patients and age-matched HCs. Flow cytometry analysis revealed that the frequencies of NK cells expressing the inhibitory receptors TIGIT and PD-1 were significantly higher in DLBCL patients than in HCs (**Figures 1A, D**). In contrast, the expression level of the active receptor CD226 on NK cells was reduced in DLBCL patients compared to HCs (**Figure 1B**). No significant difference was observed in the expression level of CD96 on NK cells between the two groups (**Figure 1C**). We further determined TIGIT and its family molecules on the CD3^-^CD56^dim^NK cell subpopulation, a mature and highly cytotoxic subset. The results were consistent with those observed in total NK cells. The expression levels of TIGIT and PD-1 were higher, while the expression level of CD226 was lower on CD3^-^CD56^dim^NK cells from DLBCL patients than those of HC. No significant difference was found in CD96 expression on CD3^-^CD56^dim^NK cells between the two groups (**Figures 1D–G**) (**Table 2**).

**Table 2:** TIGIT, CD226, CD96 and PD-1 expression on NK cells, T cells, and NKT cells in DLBCL and HCs.

	DLBCL (n=30)	HC (n=25)	P	Significance
TIGIT^+^NK (%)	26.44 ± 9.379	19.63 ± 8.45	0.007	**
CD226^+^NK (%)	71.76 ± 15.94	90.28 ± 6.777	<0.0001	****
CD96^+^NK (%)	17.58 ± 7.43	20.06 ± 4.933	0.1595	ns
PD-1^+^NK (%)	5.737 ± 10.17	1.575 ± 1.597	0.0481	*
TIGIT^+^CD3^-^CD56^dim^ NK (%)	26.96 ± 10.10	19.25 ± 7.524	0.0027	**
CD226^+^CD3^-^CD56^dim^ NK (%)	72.07 ± 15.25	90.24 ± 6.561	<0.0001	****
CD96^+^CD3^-^CD56^dim^ NK (%)	17.45 ± 7.251	20.42 ± 4.878	0.0869	ns
PD-1^+^CD3^-^CD56^dim^ NK (%)	5.76 ± 8.962	1.536 ± 1.419	0.0237	*
TIGIT^+^T (%)	30.34 ± 9.748	22.52 ± 7.873	0.0021	**
CD226^+^T(%)	78.8 ± 11.29	84.59 ± 5.692	0.0237	*
CD96^+^T (%)	17.52 ± 6.157	17.19 ± 7.198	0.8528	ns
PD-1^+^T (%)	31.86 ± 11.55	20.89 ± 9.589	0.0004	***
TIGIT^+^NKT (%)	24.63 ± 7.89	22.42 ± 6.588	0.2697	ns
CD226^+^NKT(%)	78.73 ± 11.95	83.19 ± 8.397	0.1226	ns
CD96^+^NKT(%)	29.56 ± 5.289	29.4 ± 5.363	0.912	ns
PD-1^+^NKT(%)	23.46 ± 10.84	15.11 ± 8.921	0.0033	**

DLBCL, diffuse large B-cell lymphoma; HC, healthy control; *P < 0.05, **P < 0.01, ***P < 0.001, ****P < 0.0001, ns denotes not significant; data are presented as mean ± SD using an unpaired t-test.

**Figure 2:**
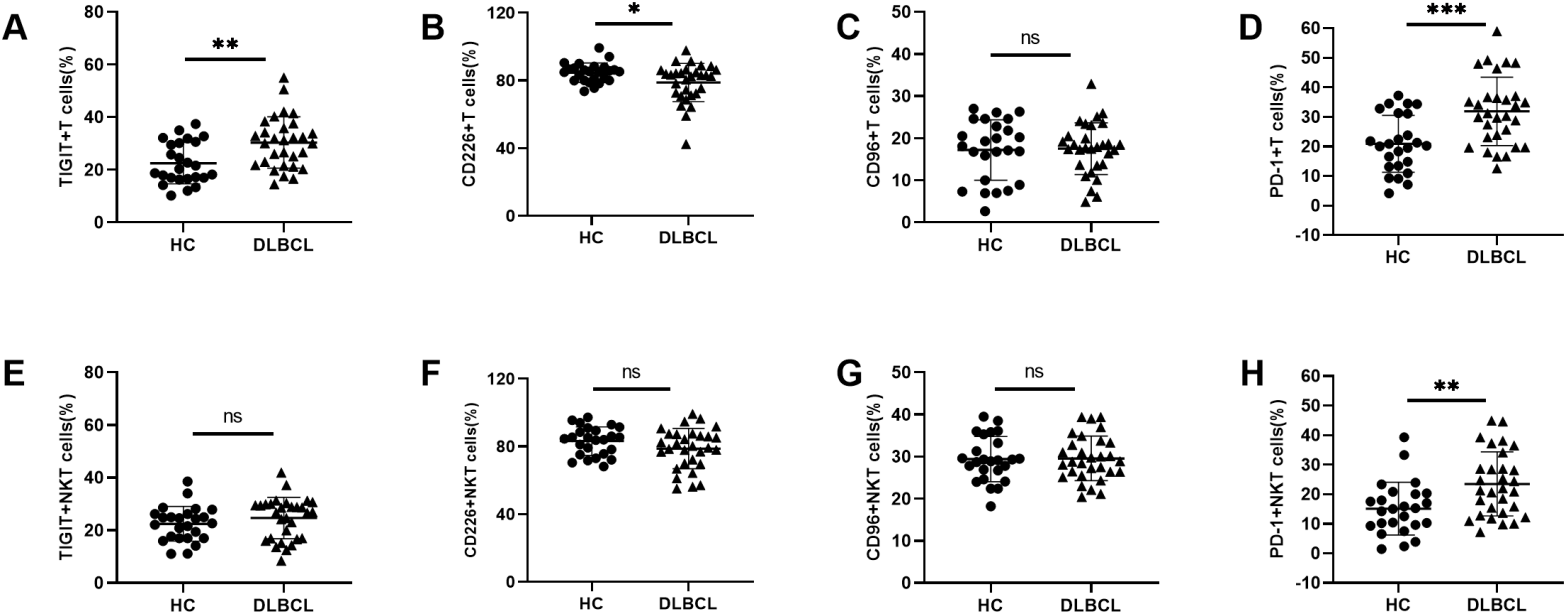
Abnormal TIGIT and its family molecules expression on T cells and NKT cells from DLBCL patients **(A–D)** Comparisons of expression levels of TIGIT, CD226, CD96, and PD-1 on T cells from DLBCL patients and HCs. **(E–H)** Comparisons of expression levels of TIGIT, CD226, CD96, and PD-1 on NKT cells from DLBCL patients and HCs. Each symbol represents the mean value from three technical replicates of one subject, and the data are shown as mean ± SD. HC, healthy control, DLBCL, diffuse B-cell lymphoma. DLBCL (n=30), HC (n=25). * indicates P<0.05, ** indicates P<0.01, *** indicates P<0.001, ns indicates no significance. Unpaired Student’s t-test was used.

Given the limited understanding of TIGIT and its family molecules on pT cells in DLBCL, we also compared their expression levels on peripheral T cells and NKT cells between DLBCL patients and HCs. Similar to the findings in NK cells, higher expression levels of TIGIT and PD-1 and lower expression level of CD226 were observed on peripheral T cells from DLBCL patients than those in HCs. No significant difference was observed in CD96 expression on T cells between the two groups (**Figures 2A–D**). For NKT cells, there were no significant differences in the expression levels of TIGIT, CD226 or CD96 between DLBCL and HCs. However, the expression level of PD-1 on peripheral NKT cells was higher in DLBCL patients than in HCs (**Figures 2E–H**) (**Table 2**).

### 3.2 Correlation of TIGIT, CD226, and PD-1 expression on NK cells in DLBCL patients

**Figure 3:**
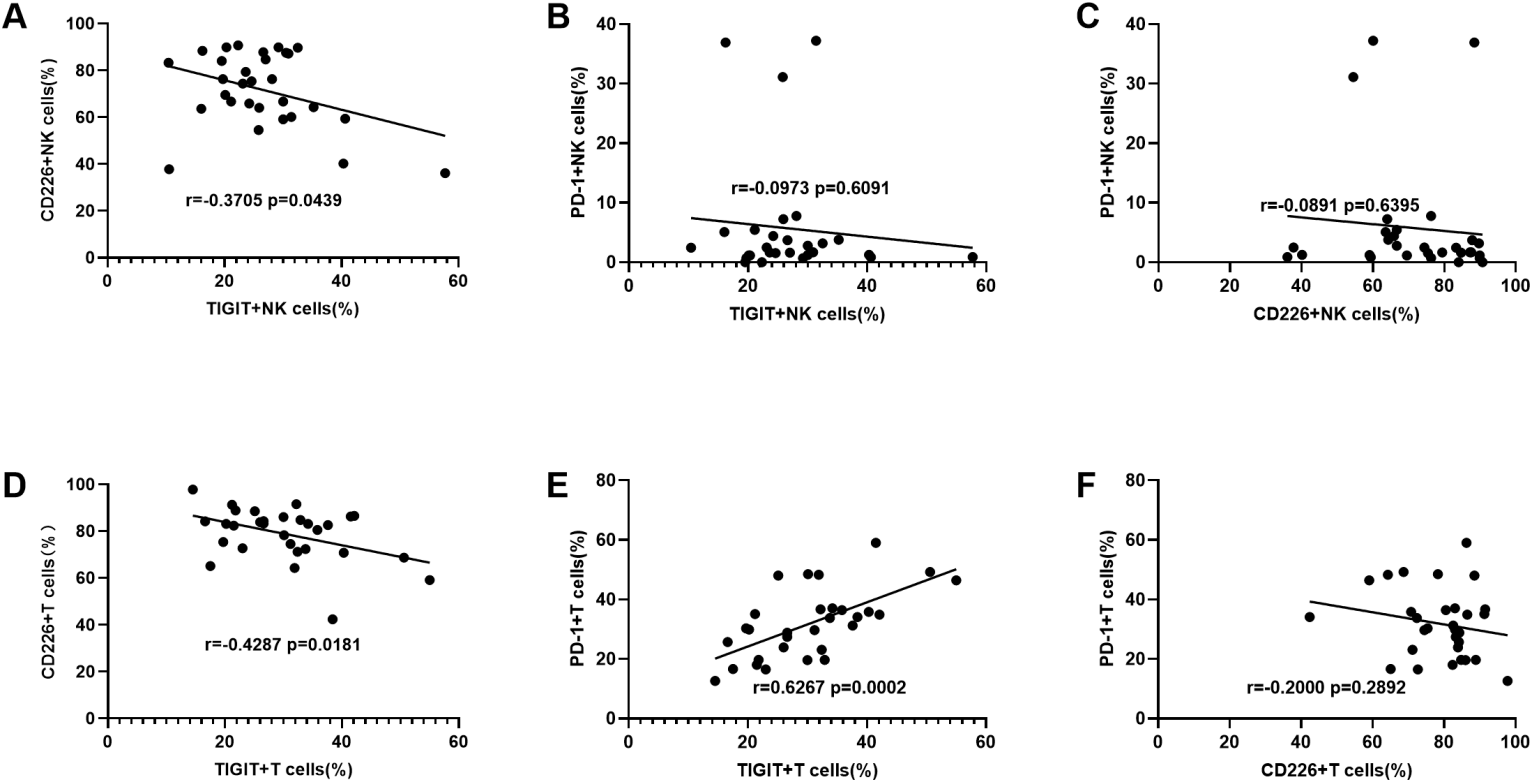
The correlation between TIGIT, CD226 and PD-1 expression levels on NK cells from DLBCL patients **(A–C)** The correlation analysis of TIGIT vs CD226, TIGIT vs PD-1, and CD226 vs PD-1 on NK cells from DLBCL patients. **(D–F)** The correlation analysis of TIGIT vs CD226, TIGIT vs PD-1, and CD226 vs PD-1 on T cells from DLBCL patients. Each symbol represents the mean value from three technical replicates of one subject. Pearson test was used for correlation analysis. Correlation coefficients (r) and p values are indicated in the figure.

Next, we analyzed the correlation between TIGIT, CD226 and PD-1 expression on peripheral NK cells and T cells in DLBCL patients. On NK cells, TIGIT expression was negatively correlated with CD226 expression but showed no significant correlation with PD-1 expression. Additionally, no significant correlation was observed between CD226 and PD-1 expression on NK cells (**Figures 3A–C**). On T cells, TIGIT expression was negatively correlated with CD226 expression and positively correlated with PD-1 expression. However, no significant correlation was found between CD226 and PD-1 expression on T cells (**Figures 3D–F**).

### 3.3 Impaired NK cell function were observed in DLBCL patients

**Figure 4:**
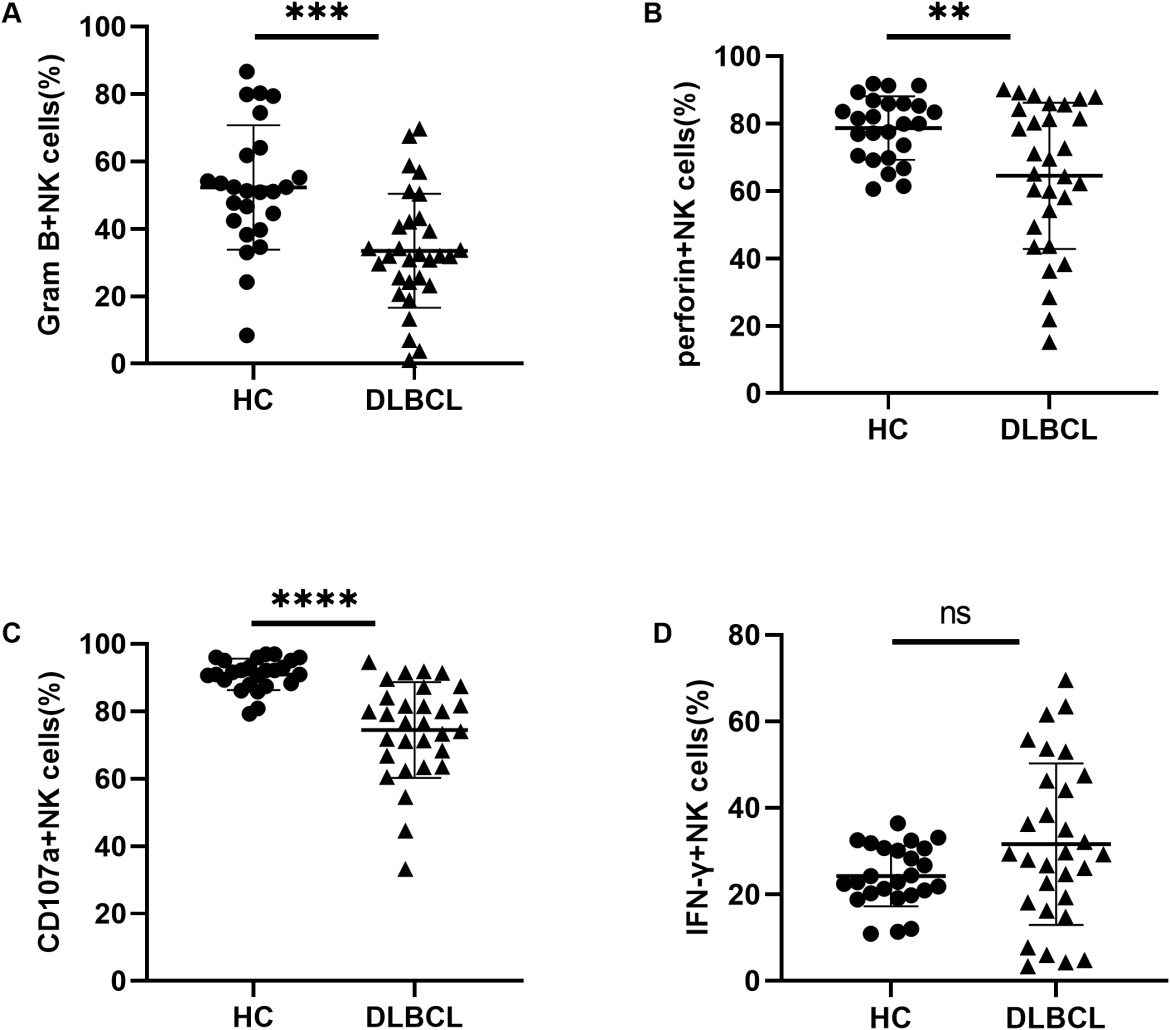
Impaired NK cell function in DLBCL patients **(A–D)** Comparisons of secretion levels of granzyme B, perforin, CD107a, and IFN-γ by NK cells from DLBCL patients and HC. Each symbol represents the mean value from three technical replicates of one subject, and the data are shown as mean ± SD. HC, healthy control, DLBCL, diffuse B-cell lymphoma. DLBCL (n=30), HC (n=25) ** indicates P<0.01, *** indicates P<0.001, **** indicates P<0.0001, ns indicates no significance. Unpaired Student’s t-test was used.

To investigate the impact of abnormal TIGIT and its family molecule expression on NK cells, we further analyzed NK cell function in DLBCL patients. We detected the ability of secreting cytokines by NK cells through flow cytometry. The results revealed that the secretion levels of granzyme B, perforin, and CD107a were significantly decreased in NK cells from DLBCL patients compared to HCs (**Figures 4A–C**). There was no significant difference in IFN-γ secretion of peripheral blood NK cells between the two groups (**Figure 4D**). These findings suggest that NK cell activity is impaired in DLBCL patients, potentially leading to reduced cytotoxicity against target cells.

### 3.4 Enhanced tumor-killing ability of NK cells was observed by blocking TIGIT

**Figure 5:**
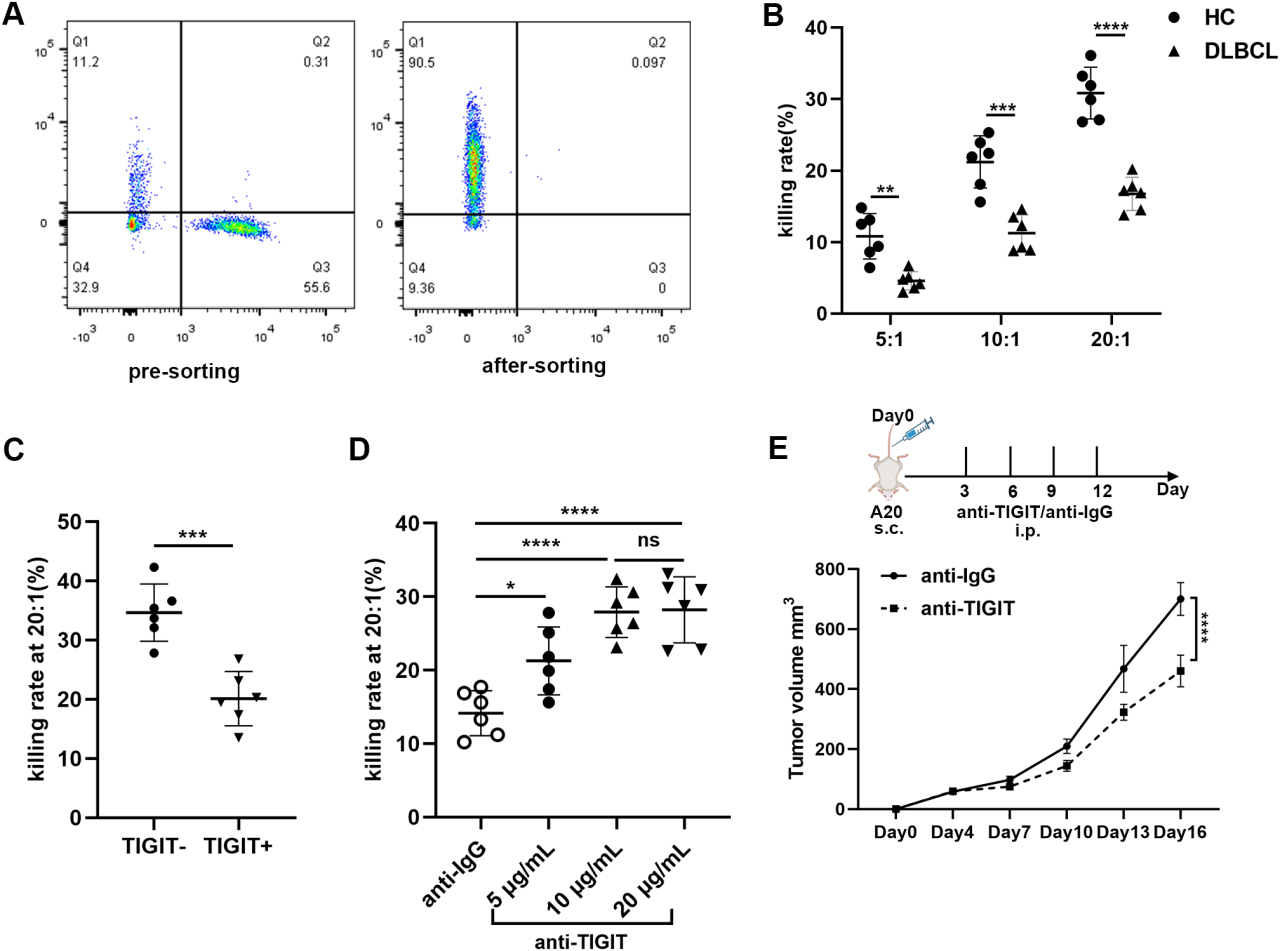
Enhanced killing ability of NK cells was observed by blocking TIGIT **(A)** Schematic representation of purity of peripheral NK cells before and after sorting. **(B)** Comparisons of the killing ability of NK cells towards DLBCL cell line SU-DHL-4 at E:T ratios of 5:1, 10:1 and 20:1 among DLBCL patients (n=6) and HC (n=6). **(C)** Comparisons of the killing ability of TIGIT^+^ NK cells and TIGIT^-^NK cells towards DLBCL cell line SU-DHL-4 at an E:T ratio of 20:1 (n=6). **(D)** Quantification of the killing ability of NK cells from DLBCL patients in the presence of varying concentrations of anti-TIGIT mAb blockade at an E:T ratio of 20:1 (n=6). **(E)** Rag2-/- mice were subcutaneously injected with 1 × 10^6^ A20 tumor cells on day0. At various time points thereafter, the mice were intraperitoneally administered either anti-TIGIT mAb or IgG as a control. Tumor volume was measured at multiple time points (n=6). Each symbol represents the mean value from three technical replicates of one subject, and the data are shown as mean ± SD. HC, healthy control. DLBCL, diffuse B-cell lymphoma. * indicates P<0.05, ** indicates P<0.01, *** indicates P<0.001, **** indicates P<0.0001, ns indicates no significance. Unpaired Student’s t-test was used **(B–D)**. Two-way analysis of variance was used **(E)**.

We evaluated the killing ability of NK cells against the DLBCL cell line SU-DHL-4 using a co-culture assay. NK cells were initially isolated from peripheral blood through magnetic bead sorting, with a purity of ≥ 90% confirmed by flow cytometry (**Figure 5A**). Compared to NK cells from HCs, those from DLBCL patients showed significantly impaired cytotoxicity at E:T ratios of 5:1, 10:1, and 20:1 (**Figure 5B**). To further investigate functional heterogeneity, TIGIT^+^ and TIGIT^-^ NK subsets were isolated by FACS. Notably, TIGIT^+^ NK cells showed markedly reduced tumor-killing efficiency compared to TIGIT^-^ NK cells (**Figure 5C**). To evaluate the therapeutic potential of TIGIT blockade, we examined its dose-dependent effects on the tumor-killing ability of NK cells. The tumor-killing ability of NK cells improved with increasing concentrations of anti-TIGIT mAb, plateauing at 10 μg/mL with no further enhancement at 20 μg/mL (**Figure 5D**). To validate these findings *in vivo*, we administered anti-TIGIT mAb to A20 tumor-bearing Rag2^-/-^ mice following the experimental scheme (**Figure 5E**). TIGIT blockade significantly suppressed tumor growth and reduced tumor mass compared to the control group (**Figure 5E**), with no observable adverse effects, indicating its safety in this model. Collectively, these results demonstrate that TIGIT inhibition effectively restore and enhance NK cell cytotoxicity in DLBCL, potentially reversing tumor-mediated immune suppression and augmenting anti-tumor immunity.

### 3.5 Elevated TIGIT expression and impaired function of NK cells in DLBCL patients with poor prognostic scores

**Figure 6:**
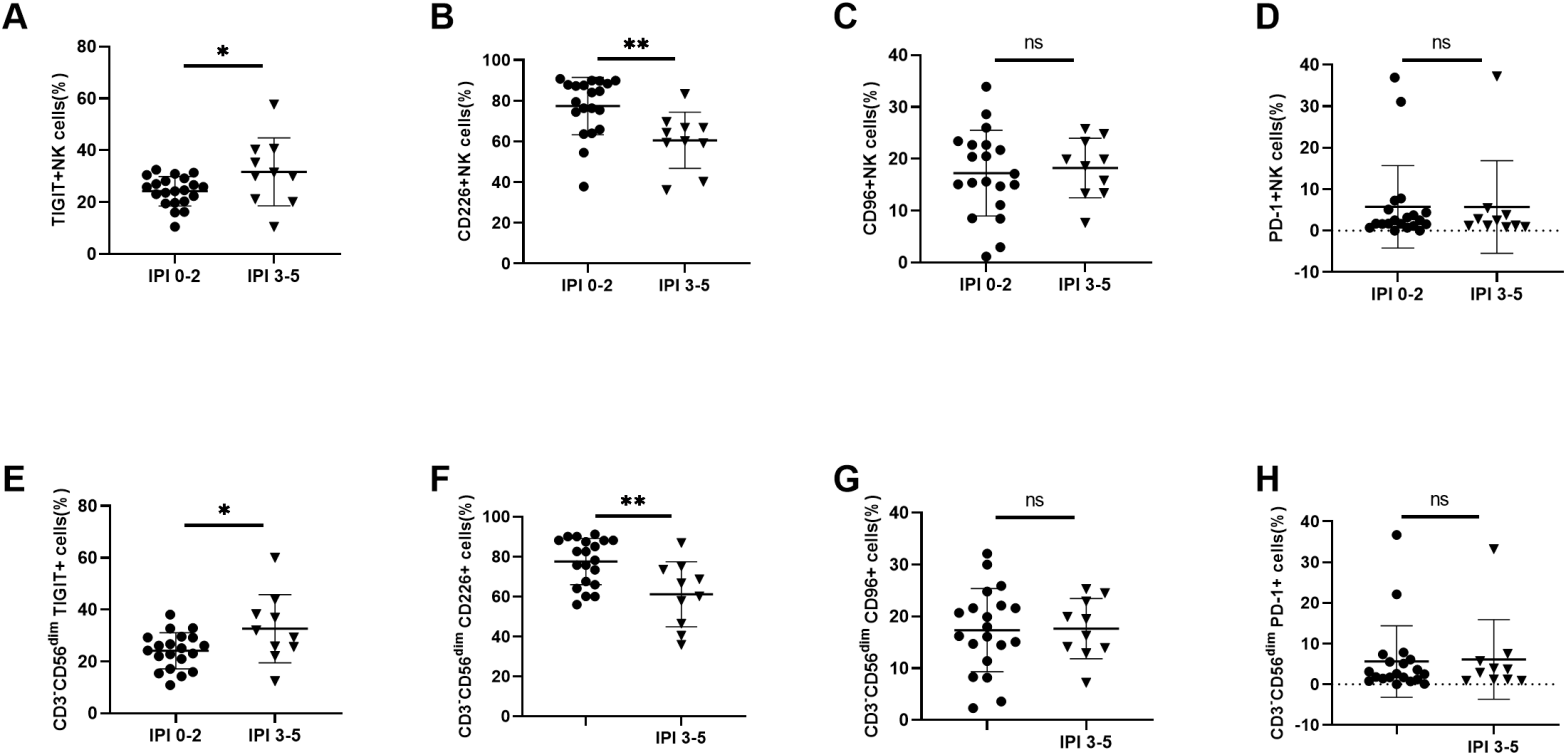
Abnormal TIGIT and its family molecules expression on NK cells in DLBCL patients with different prognostic scores **(A–D)** Comparisons of expression levels of TIGIT, CD226, CD96, and PD-1 on NK cells from IPI 0-2 and IPI 3-5 groups. **(E–H)** Comparisons of expression levels of TIGIT, CD226, CD96, and PD-1 on CD3^-^CD56^dim^NK cells from IPI 0-2 and IPI 3-5 groups. Each symbol represents the mean value from three technical replicates of one subject, and the data are shown as mean ± SD. IPI 0-2 (n=20), IPI 3-5 (n=10). * indicates P<0.05, ** indicates P<0.01, ns indicates no significance. Unpaired Student’s t-test was used.

**Figure 7:**
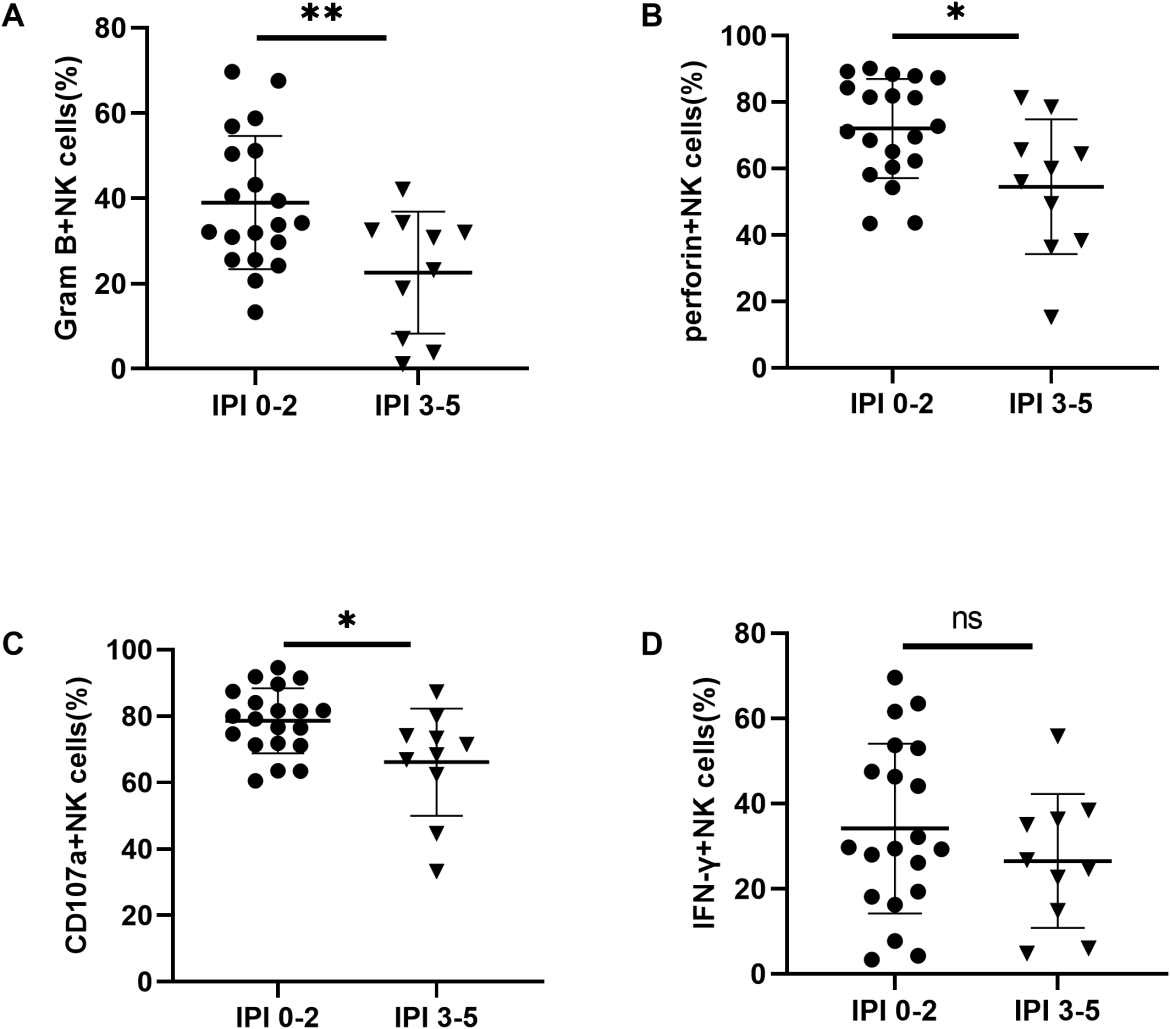
Impaired NK cell function in DLBCL patients with poor prognostic scores **(A–D)** Comparisons of secretion levels of granzyme B, perforin, CD107a, and IFN-γ by NK cells from IPI 0-2 and IPI 3-5 groups. Each symbol represents the mean value from three technical replicates of one subject, and the data are shown as mean ± SD. IPI 0-2 (n=20), IPI 3-5 (n=10). * indicates P<0.05, ** indicates P<0.01, ns indicates no significance. Unpaired Student’s t-test was used.

To assess whether the frequency, subsets, and functions of NK cells differ among DLBCL patients with varying prognostic risks, we stratified patients into two groups based on their IPI scores: IPI 0-2 (low-risk) and IPI 3-5 (high-risk). We found that the absolute number of NK cells was significantly lower in the IPI 3-5 group compared to the IPI 0-2 group, primarily due to a reduction in CD56^dim^ NK cells (**Supplementary Figures S3B, D**). The percentage of NK cells in lymphocyte cells was also decreased in the IPI 3-5 group although with no significant difference (**Supplementary Figure S3A**). We further determined the expression of TIGIT and its family molecules, including TIGIT, CD226, CD96 and PD-1, on NK cells and CD56^dim^ NK cells from IPI 3-5 group and IPI 0-2 group. Flow cytometry analysis revealed that TIGIT expression on NK cells was significantly higher in the IPI 3-5 group than in the IPI 0-2 group (**Figure 6A**), whereas CD226 expression on NK cells was lower in the IPI 3-5 group (**Figure 6B**). No significant differences were observed in the expression levels of PD-1 or CD96 on NK cells between the two groups (**Figures 6C, D**). Similar trends were observed in the CD3^-^CD56^dim^ NK cell subset, with higher TIGIT expression and lower CD226 expression in the IPI 3-5 group, while PD-1 and CD96 levels remained unchanged (**Figures 6E–H**) (**Table 3**). Moreover, the secretion levels of granzyme B, perforin, and CD107a were decreased in NK cells from DLBCL patients with high-risk IPI scores (**Figures 7A–C**). There was no significant difference in IFN-γ secretion of peripheral NK cells between the two groups (**Figure 7D**).

**Table 3:** TIGIT, CD226, CD96 and PD-1 expression on NK cells and CD3^-^CD56^dim^ NK cells in DLBCL patients.

	IPI (0-2) (n=20)	IPI (3-5) (n=10)	P	Significance
TIGIT^+^NK (%)	24.19 ± 1.241	31.68 ± 13.11	0.0327	*
CD226^+^NK (%)	77.37 ± 14.1	60.54 ± 13.76	0.0043	**
CD96^+^NK (%)	17.25 ± 8.264	18.25 ± 5.744	0.7353	ns
PD-1^+^NK (%)	5.756 ± 9.938	5.701 ± 11.17	0.9892	ns
TIGIT^+^CD3^-^CD56^dim^ NK (%)	24.13 ± 6.979	32.62 ± 13.14	0.0272	*
CD226^+^CD3^-^CD56^dim^ NK (%)	77.53 ± 11.6	61.16 ± 16.32	0.0036	**
CD96^+^CD3^-^CD56^dim^ NK (%)	17.36 ± 8.022	17.65 ± 5.788	0.9182	ns
PD-1^+^CD3^-^CD56^dim^ NK (%)	5.592 ± 8.787	6.098 ± 9.777	0.887	ns

DLBCL, diffuse large B-cell lymphoma. *P < 0.05, **P < 0.01, ns denotes not significant; data are presented as mean ± SD using an unpaired t-test.

## 4 Discussion

NK cells are key players in the immune system, playing a critical role in anti-tumor immunity. As essential components of the innate immune system, NK cells share functional similarities with cytotoxic CD8^+^ T cells, as both can directly kill tumor cells and secrete cytokines to modulate adaptive immune responses (33, 34). However, the quantity and functional status of NK cells in DLBCL patients remain poorly understood. In this study, we observed significant reductions in both the proportion and function of NK cells in DLBCL patients, accompanied by dysregulated expression of immune checkpoint molecules. Specifically, TIGIT and PD-1 were upregulated, while CD226 was downregulated on NK cells, contributing to NK cell dysfunction and tumor immune evasion. Notably, TIGIT blockade effectively restored NK cell cytotoxicity in both *in vivo* and *in vitro* models. Furthermore, DLBCL patients with high-risk prognostic scores exhibited higher TIGIT and lower CD226 expression, which correlated with more severe NK cell impairment, suggesting a potential role of these molecules in disease progression.

Consistent with previous studies reporting reduced NK cell percentages in colorectal and hepatocellular carcinomas (35, 36), our study revealed decreased proportions and absolute numbers of peripheral NK cells in DLBCL patients compared to HCs, suggesting an insufficient anti-tumor effector population. Notably, the CD56^dim^ subset, which exhibits potent cytotoxicity against tumors, was particularly reduced—a finding consistent with observations in follicular lymphoma (37). These quantitative deficits likely impair tumor control and contribute to DLBCL progression. However, it is worth noting that reduced peripheral NK cell counts may reflect increased trafficking to tumor tissues and adjacent normal tissues rather than an overall depletion (38). Furthermore, the causal relationship between NK cell deficiency and DLBCL remains unclear, as it is unknown whether this drives disease pathogenesis or results from tumor-induced immunosuppression, such as the production of immune-suppressive cytokines. Thus, future studies should investigate NK cell distribution across tissue compartments and perform longitudinal analyses of NK cell dynamics during treatment to elucidate their relationship with tumor burden.

The function of NK cells is regulated by a delicate balance between activating and inhibitory signals (39). Surface receptors on NK cells, including both HLA-specific receptors and non-HLA-specific receptors, play a crucial role in maintaining their normal functionality (12, 40). Our study reveals increased expression of TIGIT and PD-1, along with decreased CD226 expression, on NK cells (including CD56^dim^ NK cells) in DLBCL patients. Current evidence suggests that TIGIT upregulation in malignancies involves multiple mechanisms, such as dysregulated cytokine signaling (e.g., IL-6/STAT3), chronic stimulation by its ligand CD155, and epigenetic modifications like hypomethylation (25, 41, 42). Thus, further studies are needed to elucidate the specific mechanisms driving TIGIT upregulation in DLBCL. Additionally, we found no significant difference in CD96 expression on NK cells between DLBCL patients and HCs. CD96 exhibits dual functionality, with varying effects across different tumor microenvironments. Its expression is closely associated with immune infiltration, indicating that localized changes within the tumor microenvironment may not be fully reflected in peripheral NK cell analyses (43). Therefore, future studies should focus on characterizing intratumoral NK cells and investigating how CD96 expression influences their functional outcomes in DLBCL.

Additionally, a negative correlation between the TIGIT and CD226 expression on NK cells was observed in DLBCL patients, further impairing NK cell activity. This negative correlation may be attributed to several factors. Compared to CD226, TIGIT exhibits a higher affinity for shared ligand binding (44). Mechanistically, TIGIT can inhibit CD226 function through multiple pathways. For instance, the cis-interaction between TIGIT and CD226 physically interferes with PVR-CD226 interactions and prevents CD226 homodimerization, thereby inhibiting its activation. Furthermore, TIGIT, in concert with PD-1,could suppress CD226 phosphorylation and impair immune function (45).

Upon TIGIT binding to its ligands, TIGIT signaling suppresses NK cell function through Grb2/SHIP1-mediated inhibition of PI3K/MAPK pathways and β-Arrestin 2-mediated suppression of NF-κB pathways (46, 47). To explore the clinical implications of dysregulated TIGIT and its family molecules, we assessed of NK cell cytotoxicity in DLBCL patients. Our results demonstrated significant functional impairment in NK cells, characterized by decreased secretion of cytotoxic molecules (perforin, granzyme B, and CD107a) and diminished tumor-killing capacity. Interestingly, IFN-γ secretion levels remained largely unchanged, suggesting distinct regulatory mechanisms for IFN-γ compared to other NK cell effector molecules. Given that CD56^bright^ cells produce significantly more IFN-γ per cell than CD56^dim^ cells, it is possible that the remaining CD56^bright^ NK cells compensate for the loss by increasing their IFN-γ production (48). However, the precise mechanisms underlying sustained IFN-γ levels remains unclear and require further investigation.

The application of immune checkpoint inhibitors, particularly PD-1 and CTLA-4 inhibitors, has demonstrated significant efficacy in treating malignancies (18). Similarly, TIGIT blockade has been shown to enhance T cell function in both cancer and infectious diseases (49–51). In this study, we demonstrated that TIGIT blockade enhances of NK cell cytotoxicity against DLBCL cells in both *in vitro* and *in vivo* models. These findings provide a strong theoretical foundation and highlight the translational potential of TIGIT inhibitors as a novel therapeutic strategy for DLBCL, especially in NK cell-based immunotherapy. However, our *in vitro* experiments revealed that the tumor-killing ability of NK cells plateaued at 10 μg/mL, with no further enhancement at 20 μg/mL. This aligns with a previous study by Wang et al. in prostate cancer, suggesting that TIGIT binding sites on NK cells may become saturated at this concentration (52). Therefore, TIGIT blockade alone has a limited therapeutic ceiling, emphasizing the need to explore combination therapies with other immune checkpoint inhibitors to maximize NK cell-mediated anti-tumor responses. Additionally, strategies to enhance CD226 expression in combination with TIGIT blockade warrant further investigation.

Moreover, we analyzed changes in the quantity and function of NK cells among DLBCL patients stratified by prognostic risk. Notably, patients with high-risk prognoses exhibited more significant reductions in NK cell numbers and functionality. Specifically, these patients showed higher frequencies of TIGIT expression and lower frequencies of CD226 expression on NK cells, which correlated with further impairment in cytotoxic activity. These findings suggest that TIGIT expression levels may be closely associated with DLBCL prognostic risk, underscoring its potential as a prognostic biomarker. However, establishing TIGIT as an independent prognostic factor will require long-term outcome data, including overall survival (OS) and progression-free survival (PFS). Future studies should extend follow-up periods and integrate comprehensive clinical data to perform robust multivariate analyses. Such efforts will help validate the prognostic significance of TIGIT in DLBCL and explore potential interactions between TIGIT and other clinical features.

Altogether, our findings reveal significant alterations in both the quality and quantity of NK cells in DLBCL patients, underscoring the critical role of TIGIT in DLBCL pathogenesis. This results establish a robust foundation for targeting TIGIT as a potential diagnostic and therapeutic target, paving the way for developing novel immunotherapies aimed at restoring NK cell functions through TIGIT blockade. However, our study has certain limitations. Although the sample size was sufficient to identify major trends, it may limit the ability to perform detailed subgroup analyses or detect more subtle effects. Additionally, despite achieving ≥90% purity in isolated NK cells, residual cell types could still influence experimental accuracy. To advance this research, several improvements should be considered. Expanding sample sizes and establishing multicenter collaborations would enhance the generalizability of findings across diverse patient populations. Employing advanced cell isolation technologies could further improve NK cell purity. Moreover, mechanistic studies are needed to fully elucidate how TIGIT-mediated signaling pathways suppress NK cell function in DLBCL, potentially uncovering new therapeutic targets and refine existing strategies. In conclusion, targeting TIGIT represents a promising NK cell-based immunotherapy strategy for DLBCL, with the potential to transform the therapeutic landscape of this disease and beyond.

## Data Availability

The original contributions presented in the study are included in the article/**Supplementary Material**. Further inquiries can be directed to the corresponding authors.
