# Supplementary material for: Increased TIGIT expression correlates with impaired NK cell function in diffuse large B-cell lymphoma
Source: Front Oncol. 2025 Mar 31;15:1551061. doi: 10.3389/fonc.2025.1551061 (PMC11994634; doi:10.3389/fonc.2025.1551061)
Supplement: Supplementary file 1 [file DataSheet1.docx]

Supplementary Material


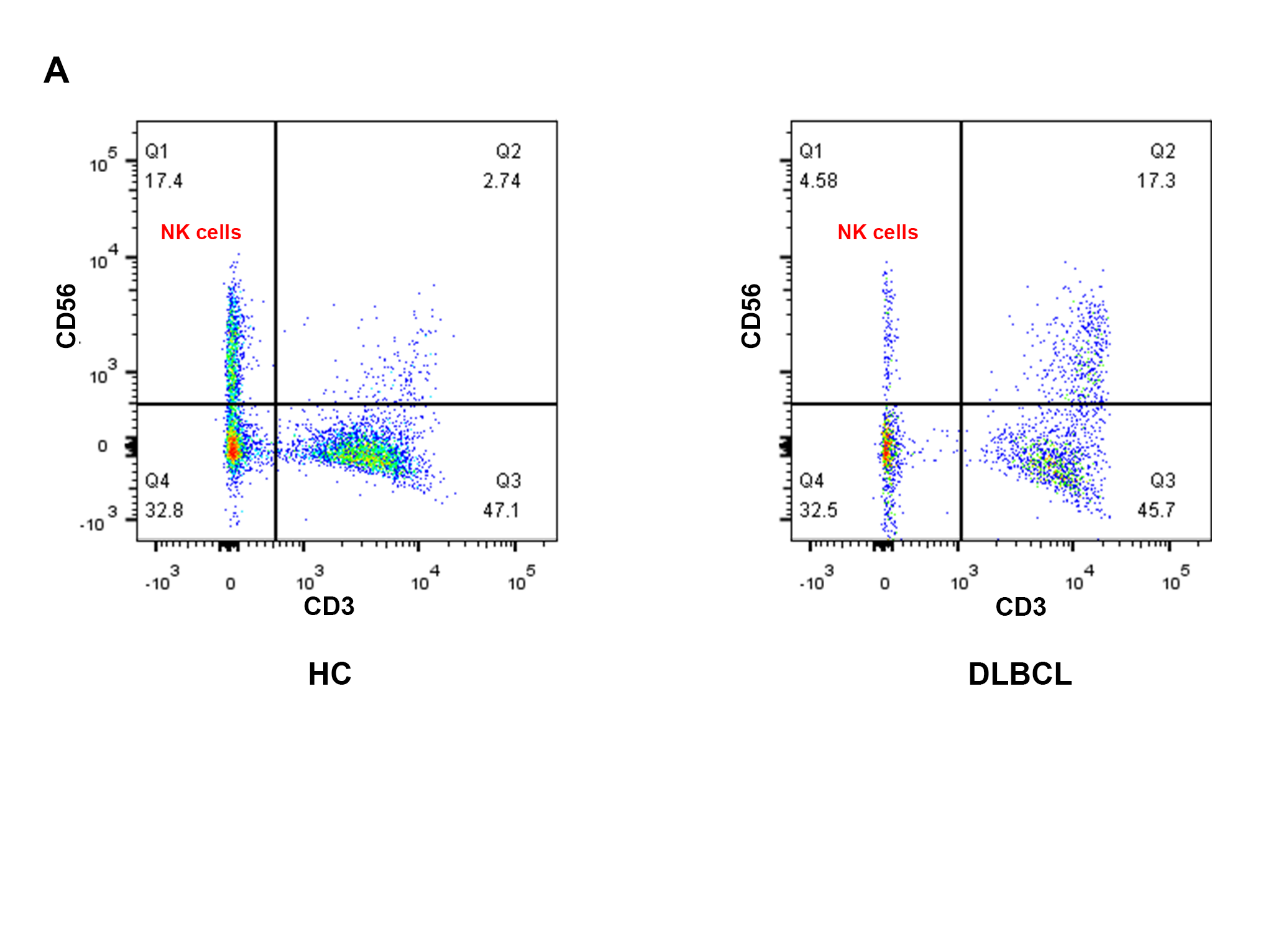


**FigureS1 (A)** Schematic representation of the proportion of NK cells in peripheral blood lymphocytes from DLBCL patients and HC. HC, healthy control. DLBCL, diffuse B-cell lymphoma.


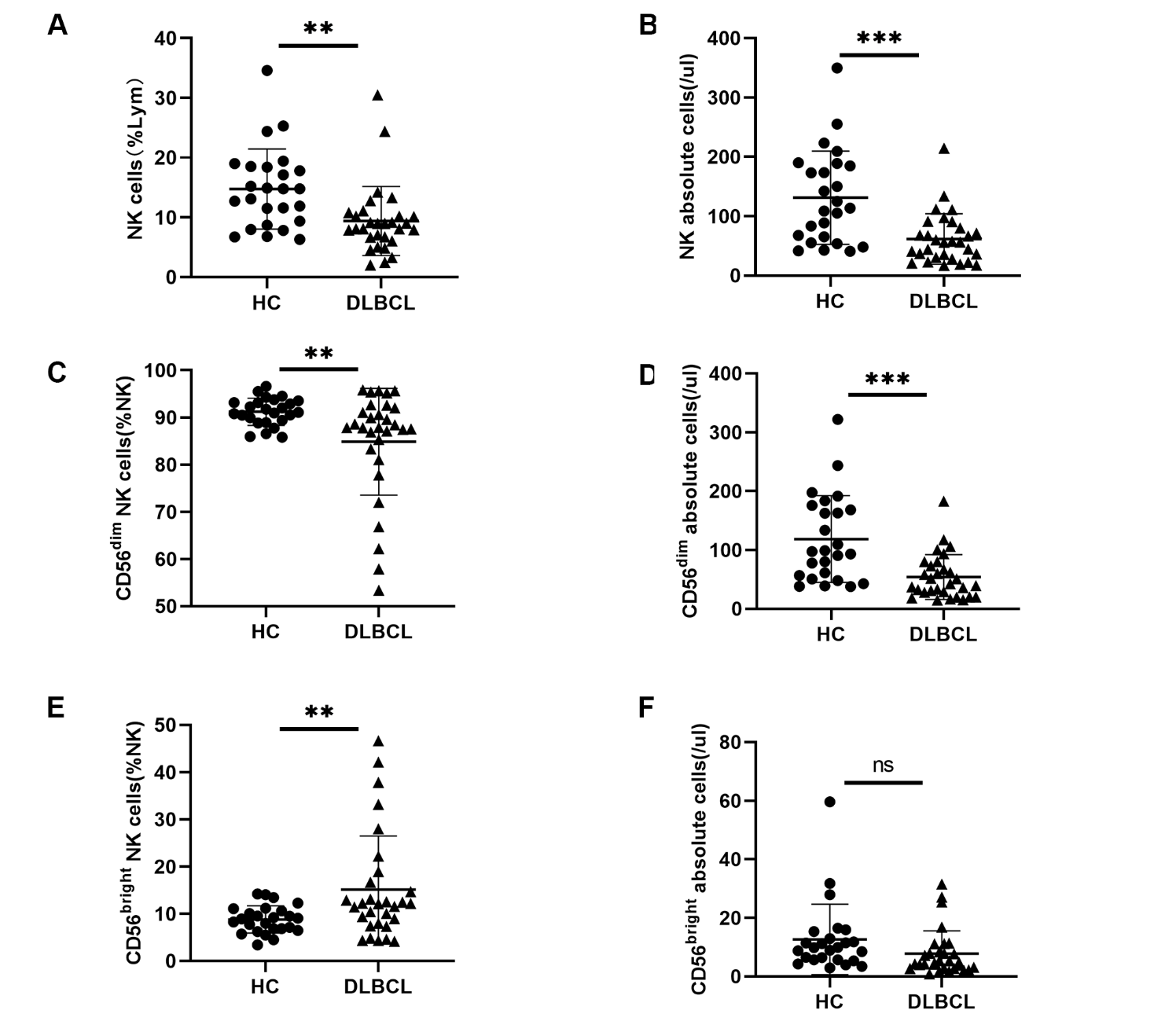


**FigureS2 Comparison of NK cell proportion and absolute numbers between HC and DLBCL patients. (A)** Comparison of the proportion of NK cells in peripheral blood lymphocytes between DLBCL patients and HC. **(B)** Comparison of the NK cell absolute number between DLBCL patients and HC. **(C)** Comparison of the proportion of CD56^dim^ NK cells within NK cells between DLBCL patients and HC. **(D)** Comparison of the CD56^dim^ NK cell absolute number between DLBCL patients and HC. **(E)** Comparison of the proportion of CD56^bright^ NK cells within NK cells between DLBCL patients and HC. **(F)** Comparison of the CD56^bright^ NK cell absolute number between DLBCL patients and HC. Each symbol represents the mean value from three technical replicates of one subject, and the data are shown as mean ± SD. HC, healthy control. DLBCL, diffuse B-cell lymphoma. Lym, lymphocyte, DLBCL (n=30), HC (n=25). ** indicates P<0.01, *** indicates P<0.001, ns indicates no significance. Unpaired Student’s t-test was used.


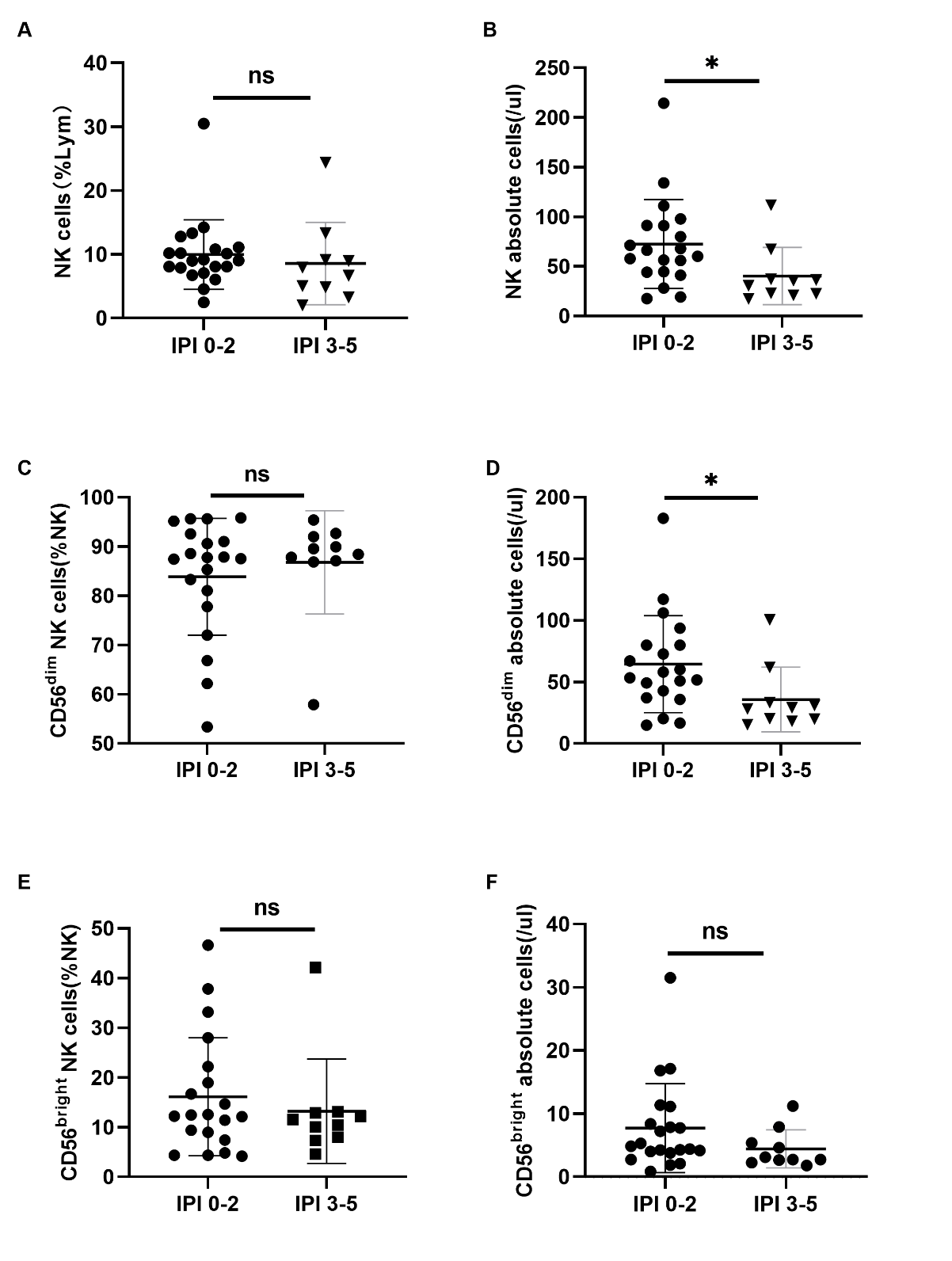


**FigureS3 Comparison of NK cell proportion and absolute numbers between DLBCL patients with different prognostic scores. (A)** Comparison of the proportion of NK cells in peripheral blood lymphocytes between IPI 0-2 and IPI 3-5 groups. **(B)** Comparison of the NK cell absolute number between DLBCL IPI 0-2 and IPI 3-5 groups. **(C)** Comparison of the proportion of CD56^dim^ NK cells within NK cells between IPI 0-2 and IPI 3-5 groups. **(D)** Comparison of the CD56^dim^ NK cell absolute number between IPI 0-2 and IPI 3-5 groups. **(E)** Comparison of the proportion of CD56^bright^ NK cells within NK cells between IPI 0-2 and IPI 3-5 groups. **(F)** Comparison of the CD56^bright^ NK cell absolute number between IPI 0-2 and IPI 3-5 groups. Each symbol represents the mean value from three technical replicates of one subject, and the data are shown as mean ± SD. IPI 0-2 (n=20), IPI 3-5 (n=10). * indicates P<0.05, ns indicates no significance. Unpaired Student’s t-test was used.

**TableS1 List of antibodies used for flow cytometric analysis**

| Antibodies | Supplier |
| --- | --- |
| anti-human CD3-PE-Cy-7 | BD Pharmingen |
| anti-human CD56-APC | BD Pharmingen |
| anti-human CD16-APC-Cy-7 | BD Pharmingen |
| anti-human TIGIT-PerCP | eBioscience |
| anti-human CD226-PE | BD Pharmingen |
| anti-human CD96-PE | BD Pharmingen |
| anti-human PD-1-FITC | BD Pharmingen |
| anti-human granzyme B-FITC | BD Pharmingen |
| anti-human perforin-PerCP | BD Pharmingen |
| anti-human CD107a-PE | BD Pharmingen |
| anti-human IFN-γ-PerCP | BD Pharmingen |
